# Cost-effectiveness of Adding Remplissage to Bankart Repair for On-track Hill-Sachs Lesions: A Markov Decision Analysis

**DOI:** 10.1177/03635465261444008

**Published:** 2026-06-01

**Authors:** Matthew Frederickson, Megan Kou, Dylan Cullinane, Mariano E. Menendez

**Affiliations:** * Department of Orthopaedic Surgery, University of California, Davis, Sacramento, California, USA; † College of Medicine, California Northstate University, Elk Grove, California, USA; ‡ University of California, Los Angeles, Los Angeles, California, USA; Investigation performed at the University of California, Davis Medical Center, Sacramento, California, USA

**Keywords:** Markov, Monte Carlo analysis, probabilistic sensitivity analysis, cost-effectiveness, remplissage, on-track Hill-Sachs

## Abstract

**Background:**

Remplissage decreases recurrence after arthroscopic Bankart repair for anterior shoulder instability but has historically been reserved for off-track Hill-Sachs lesions. The addition of remplissage for patients with on-track lesions remains controversial because of concerns about added operative costs and potential loss of range of motion. The cost-effectiveness of remplissage in patients with on-track Hill-Sachs lesions has not been established.

**Purpose:**

To evaluate the cost-effectiveness of arthroscopic Bankart repair with remplissage versus isolated Bankart repair in patients with on-track Hill-Sachs lesions.

**Study Design:**

Economic decision analysis; Level of evidence, 3.

**Methods:**

A Markov decision model simulated a 10-year period from a societal perspective. There were 2 treatment strategies compared: isolated arthroscopic Bankart repair (IBR) and arthroscopic Bankart repair with remplissage (BR+R). Health states included stable shoulder, recurrent instability, stable shoulder after revision, and persistent instability. Transition probabilities were derived from random-effects meta-analysis of recurrence rates at a median 34.7-month follow-up. Costs (in 2025 United States dollars) included direct medical costs and indirect costs related to productivity loss. Costs and quality-adjusted life-years (QALYs) were discounted at 3% annually. Outcomes included total costs, QALYs, net monetary benefit (NMB), and incremental cost-effectiveness ratios (ICERs). Probabilistic sensitivity analysis (PSA) with Monte Carlo microsimulation was conducted with 1000 simulated patients and 10,000 PSA iterations. One-way sensitivity analysis evaluated the influence of individual parameters. Cost-effectiveness acceptability curves were generated at willingness-to-pay (WTP) thresholds of $50,000/QALY and $100,000/QALY.

**Results:**

Over a 10-year time horizon, BR+R yielded 8.59 QALYs at a cost of $24,980, whereas IBR yielded 8.18 QALYs at a cost of $23,333. BR+R was cost-effective in 99.89% and 99.99% of simulations at WTP thresholds of $50,000/QALY and $100,000/QALY, respectively. One-way sensitivity analysis showed that the model was most sensitive to recurrence probabilities.

**Conclusion:**

For patients with on-track Hill-Sachs lesions and subcritical glenoid bone loss, Bankart repair with remplissage was cost-effective compared with isolated Bankart repair, driven by reductions in recurrence and revision surgery. These findings support the consideration of remplissage in patients with on-track lesions, particularly those at a higher risk for recurrence.

Arthroscopic Bankart repair is generally considered the preferred first-line surgical treatment for anterior shoulder instability with subcritical glenoid bone loss (GBL).^39^ However, recurrence after this procedure is not uncommon, with a higher risk in younger patients, athletes, and those with GBL or Hill-Sachs lesions.^12,49^ Remplissage has emerged as an adjunctive procedure to arthroscopic Bankart repair for patients with Hill-Sachs lesions and subcritical GBL,^39^ resulting in significant reductions of recurrent instability compared with isolated Bankart repair in this population.^24^ The decision to add remplissage is primarily informed by the glenoid track concept, which classifies Hill-Sachs lesions as “on-track” or “off-track” based on whether the medial margin of the defect remains contained within the functional glenoid track during motion.^13,14,45^ While there is a general consensus that off-track lesions benefit from remplissage because of a high engagement risk, the role of remplissage for on-track Hill-Sachs lesions remains controversial.^29,32,39^ Within the on-track category, contact or overhead athletes and patients with so-called “near-track” lesions, those that are approaching the threshold for off-track classification, are at a higher risk and may benefit from the addition of remplissage.^3,27,55^ There is growing clinical interest in remplissage for on-track lesions, with emerging evidence demonstrating lower recurrent instability and improved clinical outcomes in selected higher risk patients compared with isolated Bankart repair.^9,22,47^ A recent systematic review by Villarreal-Espinosa et al^50^ of 537 patients with on-track lesions and subcritical GBL reported lower recurrent instability after Bankart repair with remplissage compared with isolated Bankart repair (odds ratio [OR], 0.22 [95% CI, 0.09-0.53]), with subgroup analyses suggesting a particular benefit in high-risk groups.

Despite growing clinical evidence favoring remplissage for selected patients with on-track lesions, the decision to add remplissage requires the consideration of added operative costs and time, additional implant utilization, and uncertainty regarding long-term sequelae such as the progression of glenohumeral osteoarthritis due to altered joint anatomy and biomechanics.^1,2,18,26,31,43^ As health care systems increasingly emphasize value-based care, economic evidence is essential to determine whether the higher upfront costs of adjunctive procedures translate to long-term value. Recent break-even cost analysis has shown that while Bankart repair with remplissage requires higher upfront costs, it is financially justified for off-track lesions, with variable economic viability for on-track lesions depending on institutional cost parameters.^43^ However, break-even analyses do not model economic outcomes over extended time horizons or account for differences in patients’ quality of life. Markov decision analytic modeling provides this comprehensive evaluation by simulating outcomes over extended periods and incorporating both clinical events and quality-adjusted outcomes.^46^ To our knowledge, this approach has not yet been applied to remplissage.

The purpose of this study was to evaluate the cost-effectiveness of arthroscopic Bankart repair with remplissage versus isolated Bankart repair in patients with on-track Hill-Sachs lesions. Given recent evidence demonstrating lower recurrent dislocation rates with remplissage compared to isolated Bankart repair in patients with on-track lesions,^50^ we hypothesized that Bankart repair with remplissage would be cost-effective compared to isolated Bankart repair.

## Methods

### Study Design

A Markov model is a mathematical framework that can be used in medical decision-making to model long-term outcomes and cost-effectiveness based on the probability of patients transitioning among defined health states. Each health state is associated with a cost, health utility, and transition probability that determines a patient's movement between states over successive time cycles.^46^ Model parameters are derived from published clinical and economic studies and, where necessary, assumption-based estimates informed by expert opinion. A key economic output of a Markov model includes the total quality-adjusted life-years (QALYs), which measure both the quality and quantity of life lived in different health states over time.^53^

This study modeled a hypothetical cohort of young, active patients with anterior shoulder instability, on-track Hill-Sachs lesions, and subcritical GBL (<20%) undergoing primary arthroscopic stabilization. Patient age and degree of GBL were not modeled as independent variables but were reflected through the source data, which included patients with a mean age of approximately 26 years and GBL ranging from 2.7% to 20%.^50^ Markov model–based cost-effectiveness analysis was conducted to compare Bankart repair with remplissage versus isolated Bankart repair for the management of on-track Hill-Sachs lesions. The model was developed using TreeAge Pro Healthcare (Version 2025; TreeAge Software) to simulate long-term clinical and economic outcomes from a societal perspective, incorporating both direct medical costs and indirect costs related to productivity loss. The primary outcome was the incremental cost-effectiveness ratio (ICER), expressed as the additional cost per QALY gained with Bankart repair with remplissage compared with isolated Bankart repair.^4^ In this model, both costs and QALYs were discounted at a rate of 3% annually in accordance with recommendations from the Second Panel on Cost-Effectiveness in Health and Medicine.^41^ Institutional review board approval was not required.

### Model Structure

**Figure 1.**
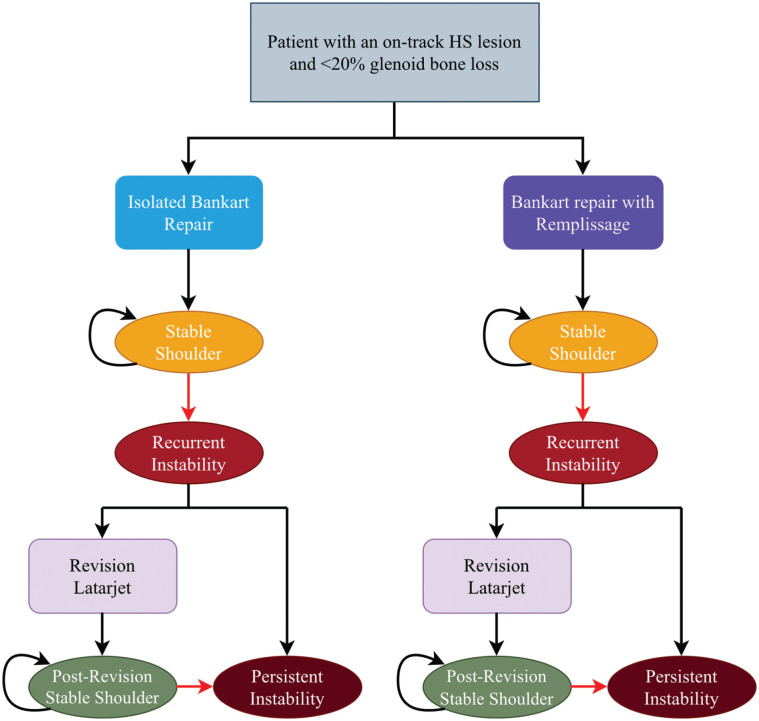
Markov model flow diagram depicting the 2 treatment strategies: isolated Bankart repair (IBR) versus Bankart repair with remplissage (BR+R). Patients began in the stable shoulder state after their index procedure and could transition to recurrent instability based on procedure-specific recurrence probabilities. Patients with recurrent instability could either elect a revision Latarjet procedure or enter the persistent instability state. Patients who underwent a revision Latarjet procedure entered the stable shoulder after revision state but might experience recurrent instability within the first 2 post-revision cycles, at which point they entered persistent instability. Patients could undergo a maximum of 2 surgical procedures: an index procedure and one revision.

A Markov model was developed to simulate a cohort of patients undergoing arthroscopic stabilization, either isolated Bankart repair (IBR) or Bankart repair with remplissage (BR+R), after an initial shoulder dislocation. The cycle length was defined as 1 year, and the model ran for a 10-year time horizon, selected to capture the period of the highest recurrence risk, consistent with prior Markov analyses.^23,35^ The model included 4 health states: (1) stable shoulder, (2) recurrent instability, (3) stable shoulder after revision, and (4) persistent instability (Figure 1). Patients occupied 1 health state per cycle and could transition between states at the beginning of each 1-year cycle. Each patient entered the model after the index procedure in the stable shoulder state. The transition from stable shoulder to recurrent instability was determined by index procedure–specific recurrence probabilities. Patients who experienced recurrent instability could elect revision surgery or continue nonoperative management. Patients who did not elect revision entered the persistent instability state for the duration of the model. Revision surgery was modeled as the open Latarjet procedure for both treatment arms, reflecting current clinical practice for revision stabilization after failed arthroscopic repair.^51^ Patients who underwent a revision Latarjet procedure entered the stable shoulder after revision state but could experience recurrent instability, modeled at a constant annual rate over the first 2 post-revision cycles based on the temporal distribution of failure after the Latarjet procedure reported by Zimmerman et al,^57^ in which all dislocations occurred within approximately 2 years.^57^ Patients who experienced recurrent instability after a revision Latarjet procedure also entered the persistent instability state for the duration of the model. Patients could undergo a maximum of 2 surgical procedures: an index procedure and one revision.

The patients who remained in the stable shoulder state accumulated health-related quality-of-life utilities, while those who remained in the recurrent instability state accumulated lower utilities because of pain and dysfunction.^11^ Because the target population was otherwise healthy, relatively young, active patients in their mid- to late-20s, the probability of background annual mortality was negligible and therefore was not modeled, which was consistent with previously published models.^21,23,30,35^ Half-cycle correction was not applied, as transitions were assumed to occur at the beginning of each cycle. This convention was applied symmetrically to both treatment arms; thus, any resulting bias did not affect the incremental comparison. Additionally, with 1-year cycle lengths over a 10-year horizon, the magnitude of any correction would be minimal relative to overall QALY estimates.^46^

### Model Inputs and Parameters

Model inputs, including costs, transition probabilities, and health state utilities, were derived from published clinical and economic studies and were selected for their direct applicability to the study population and methodological rigor (Table 1).

**Table 1:** Model Input Parameters*^*a*^*

	Base Case	PSA Range (Distribution Type)	Source
Transition probabilities, %			
Recurrence after IBR: 1-3 y	4.00	2.90-5.18 (beta)	Villarreal-Espinosa et al^50^; Do et al^16^
Recurrence after IBR: 4-10 y	2.34	1.70-3.03 (beta)	Villarreal-Espinosa et al^50^; Do et al^16^
Recurrence after BR+R: 1-3 y	0.62	0.21-1.13 (beta)	Villarreal-Espinosa et al^50^; Do et al^16^
Recurrence after BR+R: 4-10 y	0.32	0.11-0.58 (beta)	Villarreal-Espinosa et al^50^; Do et al^16^
Revision election after recurrence	48.00	34.6-61.1 (beta)	Schwihla et al^44^
Recurrence after revision Latarjet: 1-2 y	6.26	4.9-7.7 (beta)	Karavan et al^25^; Zimmermann et al^57^
Complication rates, %			
IBR	2.1	1.5-2.7 (beta)	Gonzalez-Morgado et al^20^
BR+R	0.3	0.03-0.61 (beta)	Gonzalez-Morgado et al^20^
Revision Latarjet	10.2	8.8-11.6 (beta)	Karavan et al^25^
Direct costs, $			
IBR	10,512	7890-13,399 (gamma)	Schreiner et al^43^
BR+R	14,550	10,900-18,350 (gamma)	Schreiner et al^43^
Revision Latarjet	15,308	11,539-19,339 (gamma)	Schreiner et al^43^
Physical therapy (per surgical procedure)	3480	Deterministic	Sandler et al^42^; CMS^8^
Minor complication	979	Deterministic	Sandler et al^42^; CMS^8^
Indirect costs (societal), $			
Productivity loss: index surgery	6460	Deterministic	Sandler et al^42^; BLS^6^
Productivity loss: revision Latarjet	9044	Deterministic	Sandler et al^42^; BLS^6^
Utilities (0.00-1.00)			
Recurrent instability	0.35	0.29-0.41 (normal)	Oeding et al^35^
Stable shoulder	0.91	0.85-0.97 (normal)	Oeding et al^35^
ROM loss decrement (BR+R stable shoulder only)	0.00	0.00-0.05 (one-way sensitivity analysis)	Expert opinion

a Recurrence probabilities were derived from random-effects meta-analysis of Villarreal-Espinosa et al^50^ and distributed temporally based on Do et al.^16^ Standard errors from the random-effects model were used to define beta distributions for PSA. Cost and utility distributions used standard deviations equal to 20% of the base-case value. The reported PSA ranges (10%-90%) reflect sampling across 10,000 PSA iterations. BLS, Bureau of Labor Statistics; BR+R, Bankart repair with remplissage; CMS, Centers for Medicare & Medicaid Services; IBR, isolated Bankart repair; PSA, probabilistic sensitivity analysis; ROM, range of motion.

Recurrent instability rates after IBR or BR+R were informed by a recent systematic review by Villarreal-Espinosa et al,^50^ who reported dislocation rates across 6 comparative studies over a median follow-up of 34.7 months. Pooled cumulative recurrence probabilities for each treatment arm were estimated using random-effects meta-analysis to account for differences between the included studies, weighting each study by its precision and incorporating between-study variability. For IBR, 39 recurrent dislocations occurred among 335 patients, and for BR+R, 5 recurrences occurred among 202 patients. The pooled cumulative recurrence was 11.52% for IBR (95% CI, 6.9%-17.0%; *I*^2^ = 44.3%) and 1.85% for BR+R (95% CI, 0.2%-4.6%; *I*^2^ = 0.0%) over the 2.9-year follow-up period. The standard errors from the random-effects model, computed with Python (Version 3.12), were used to define the distributions for probabilistic sensitivity analysis (PSA), providing empirically derived estimates of uncertainty.

To account for the well-documented front-loading of the recurrence risk after arthroscopic stabilization,^19,40,48^ time-varying transition probabilities were implemented across 2 periods: years 1-3 and years 4-10. The temporal distribution of recurrence was informed by Do et al,^16^ who reported that 46% of recurrence after arthroscopic Bankart repair occurred within the first 2.9 years in a cohort with a minimum 10-year follow-up that excluded patients with off-track lesions and glenoid defects >20%. Pooled 2.9-year cumulative recurrence estimates from random-effects meta-analysis of the Villarreal-Espinosa et al^50^ data (11.52% for IBR and 1.85% for BR+R) were treated as representing 46% of expected 10-year recurrence, yielding an estimated 10-year cumulative recurrence of 25.04% for IBR and 4.02% for BR+R. Subsequently, 46% of this risk was distributed in years 1-3 and 54% in years 4-10. The total recurrence risk for each period was then converted to annual probabilities using standard exponential transformation. This produced annual recurrence probabilities of 4.00% for IBR and 0.62% for BR+R during years 1-3, declining to 2.34% for IBR and 0.32% for BR+R during years 4-10.

The probability of electing revision surgery after recurrent instability was 48% based on a long-term study in which 11 of 23 patients (48%) with recurrent instability after Bankart repair for on-track Hill-Sachs lesions elected revision surgery at a mean follow-up of 124 months.^44^ The probability of recurrent instability after a revision Latarjet procedure was estimated from a systematic review by Karavan et al,^25^ who reported recurrent instability rates after a revision Latarjet procedure for failed Bankart repair across 5 studies. In a similar fashion, pooled cumulative recurrence was estimated using random-effects meta-analysis, yielding a rate of 12.14% over a mean follow-up of 48.4 months. Based on the temporal distribution of recurrence after the Latarjet procedure reported by Zimmermann et al^57^ in which all dislocations occurred within approximately 2 years after surgery, this cumulative risk was distributed equally across the first 2 post-revision cycles, yielding a constant annual rate of 6.26% within the first 2 years, with no further recurrence risk modeled thereafter.

Procedure costs were derived from published total cost ranges for IBR ($7507-$13,516), BR+R ($10,596-$18,503), and revision Latarjet procedure ($10,926-$19,690), with base-case values set as the midpoint of each range (IBR: $10,512; BR+R: $14,550; revision Latarjet: $15,308).^43^ All costs were expressed in United States dollars and followed gamma distributions with standard deviations equal to 20% of the mean.^23,35^ Indirect costs were estimated using Bureau of Labor Statistics median weekly earnings for adults aged 25 to 34 years ($1125)^6^ and the unemployment rate for the same age group (4.3%),^17^ applied as a one-time cost per surgical event using the methodology of Sandler et al.^42^ Estimated missed workdays were 30 days for index arthroscopic procedures and 42 days for revision Latarjet procedures, yielding productivity losses of $6460 and $9044, respectively. Physical therapy costs were applied as a one-time deterministic cost of $3480 per surgical episode based on 12 visits at $290 per visit, adjusted from Sandler et al^42^ using the Consumer Price Index for All Urban Consumers for medical care services.^8^ Minor complication rates were modeled as 1-cycle event probabilities: 2.1% for IBR (17/824) and 0.3% for BR+R (1/378), derived from a comparative study of complication rates after arthroscopic stabilization with and without remplissage by Gonzalez-Morgado et al,^20^ and 10.2% for revision Latarjet procedure (77/758), derived from Karavan et al.^25^ Complication events triggered a one-time cost of $979, adjusted from Sandler et al^42^ using the Consumer Price Index for All Urban Consumers for medical care services.^8^ Complication rates followed beta distributions in PSA.

Health utilities were derived from Oeding et al,^35^ who pooled Western Ontario Shoulder Instability Index (WOSI) scores from a systematic review of 19 clinical studies of anterior shoulder instability and converted them to a 0-to-1 utility scale. A utility of 0.91 was assigned to patients with a stable shoulder after surgical stabilization and 0.35 to those with recurrent instability after surgical stabilization, reflecting pooled WOSI scores from the operative cohorts of included studies.^11,37,52,56^ These values have been adopted in subsequent cost-effectiveness analyses of shoulder instability.^23^ Utilities followed normal distributions with a standard deviation of 0.05.^35^

To account for potential range-of-motion (ROM) loss associated with remplissage, a one-way sensitivity analysis was performed in which a utility decrement of 0.01 was applied to the stable shoulder state for BR+R, varying it from 0.00 to 0.05. At a decrement of 0.01, the effective utility decreased from 0.91 to 0.90. This approach was chosen because systematic reviews have demonstrated no statistically significant difference in ROM or patient-reported outcomes between BR+R and IBR, making it difficult to derive an evidence-based utility penalty.^1,31^ Sensitivity analysis instead identified the threshold decrement at which the preferred strategy would change.

### Outcome Measures

The primary outcomes obtained from the model were total costs, QALYs, and ICERs. The ICER was calculated as the difference in total costs divided by the difference in total QALYs between BR+R and IBR. Total costs represented the sum of all medical costs over the 10-year time horizon, discounted at 3% annually. QALYs were calculated by multiplying the utility weight of each health state by the total time spent in that state across all cycles.^38^

The model results were interpreted using willingness-to-pay (WTP) thresholds of $50,000 and $100,000 per QALY, consistent with commonly cited United States health economic standards.^23,34,35^ The WTP threshold represents the maximum amount that society is willing to spend for 1 additional QALY. A treatment is considered cost-effective if its ICER falls below the WTP threshold.^7^ A treatment that provides higher QALYs at a lower cost is considered dominant. When a treatment provides higher QALYs at a higher cost, the ICER determines whether the additional benefit justifies the additional expense.^10^ The net monetary benefit (NMB) was calculated at a WTP threshold of $50,000/QALY by multiplying QALYs by the WTP threshold and subtracting total costs.^36^ A higher NMB indicates greater overall value, and the treatment with the higher NMB is preferred at a given WTP threshold.

### Analyses

PSA with Monte Carlo microsimulation was performed to evaluate the impact of parameter uncertainty on model outcomes. Transition probabilities were assigned beta distributions, cost parameters were assigned gamma distributions, and utility values were assigned normal distributions, consistent with standard practice.^5,23,35^ The model performed 10,000 PSA iterations; in each iteration, parameter values were randomly sampled from their assigned distributions, and 1000 individual patients were simulated through 10 annual cycles. Aggregate outcomes for each treatment strategy included mean total costs, mean total QALYs, and the ICER. Cost-effectiveness was evaluated using ICER scatter plots and cost-effectiveness acceptability curves. One-way sensitivity analysis was conducted on the probabilities of recurrence after IBR and BR+R, as well as on the utility of recurrent instability, to evaluate the influence of individual parameter variation on model outcomes. Each parameter was varied across its plausible range while holding all other parameters at base-case values to identify threshold values at which the preferred strategy would change.

## Results

The results of PSA over a 10-year horizon are shown in Table 2. Mean total costs were $23,333 ± $2240 for IBR and $24,980 ± $2906 for BR+R, with an incremental cost of $1646 for BR+R. Mean total QALYs were 8.18 ± 0.43 for IBR and 8.59 ± 0.45 for BR+R, yielding an incremental QALY gain of 0.41 in favor of BR+R. The mean ICER for BR+R compared with IBR was $3977 per QALY gained.

**Table 2:** Probabilistic Sensitivity Analysis Results*^*a*^*

Strategy	Cost, $	Incremental Cost, $	Effectiveness, QALYs	Incremental Effectiveness, QALYs	ICER, $/QALY	NMB, $	Optimal Iterations, %
IBR	23,333 ± 2240	—	8.18 ± 0.43	—	—	385,463 ± 21,594	0.11
BR+R	24,980 ± 2906	1646	8.59 ± 0.45	0.41	3977	404,514 ± 22,718	99.89

a Data are presented as mean ± SD unless otherwise indicated. The ICER shown represents favorability for BR+R over IBR in 99.89% of iterations in this simulation. QALYs were calculated by multiplying the utility weight of each health state by the total time spent in that state across all cycles. The NMB was calculated at a willingness-to-pay (WTP) threshold of $50,000 per QALY by multiplying QALYs by the WTP threshold and subtracting total costs and shows a greater overall value of BR+R. BR+R, Bankart repair with remplissage; IBR, isolated Bankart repair; ICER, incremental cost-effectiveness ratio; NMB, net monetary benefit; QALY, quality-adjusted life-year.

**Figure 2.**
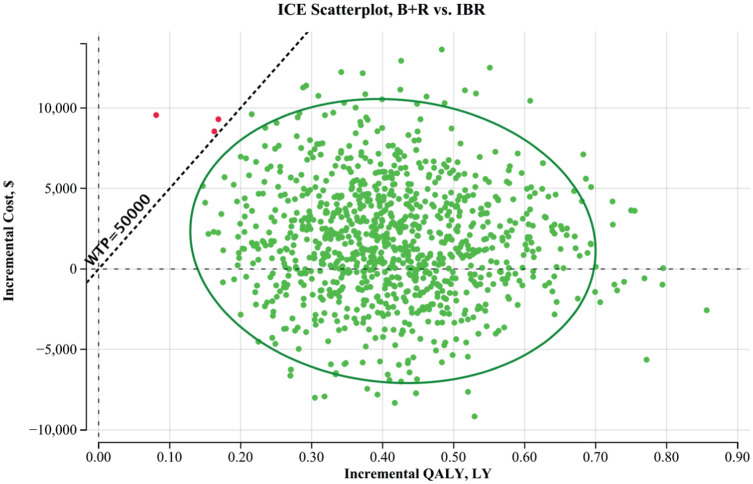
Incremental cost-effectiveness ratio scatter plot showing the first 1000 of 10,000 probabilistic sensitivity analysis (PSA) iterations. A 95% confidence ellipse is shown in green (solid ellipse), and the willingness-to-pay (WTP) threshold of $50,000 per quality-adjusted life-year (QALY) is shown as a black dashed line. Points below and to the right of the WTP line (green) represent simulations in which Bankart repair with remplissage (BR+R) was the preferred strategy. Points above and to the left (red) represent simulations in which isolated Bankart repair (IBR) was preferred. BR+R was the preferred strategy in 99.89% of simulations at the WTP threshold of $50,000/QALY.

**Figure 3.**
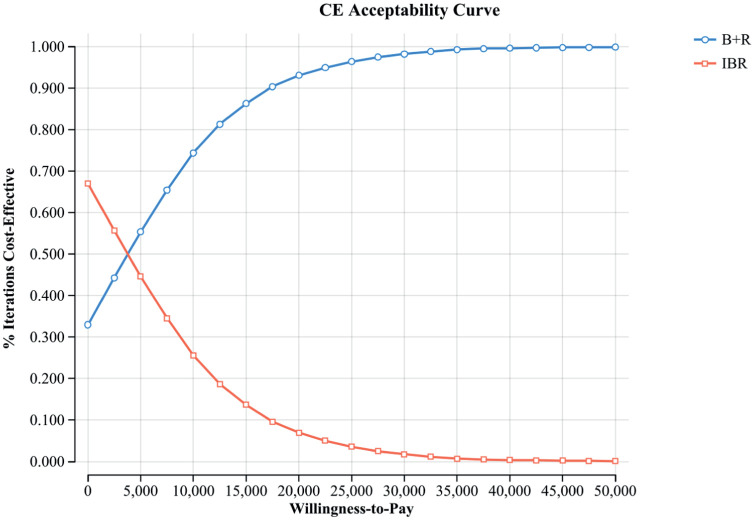
Cost-effectiveness acceptability curve comparing isolated Bankart repair (IBR; red) with Bankart repair with remplissage (BR+R; blue). The percentage of probabilistic sensitivity analysis (PSA) iterations in which each strategy was cost-effective is shown for willingness-to-pay (WTP) thresholds ranging from $0 to $50,000 per quality-adjusted life-year (QALY). BR+R was cost-effective in a higher percentage of iterations than IBR at WTP thresholds greater than $3780/QALY.

BR+R was the more cost-effective strategy at standard WTP thresholds. BR+R was the preferred strategy in 99.89% of 10,000 PSA iterations at a WTP threshold of $50,000/QALY (Figure 2). When the WTP threshold was increased to $100,000/QALY, BR+R was preferred in 99.99% of simulations. The cost-effectiveness acceptability curve (Figure 3) demonstrated that IBR was preferred only at WTP thresholds below $3780/QALY. The cost-effectiveness of BR+R over IBR was further assessed using the NMB at a WTP threshold of $50,000. BR+R had a higher NMB of $404,514 ± $22,718 compared with IBR at $385,463 ± $21,594, representing a net benefit of $19,051 in favor of BR+R.

**Figure 4.**
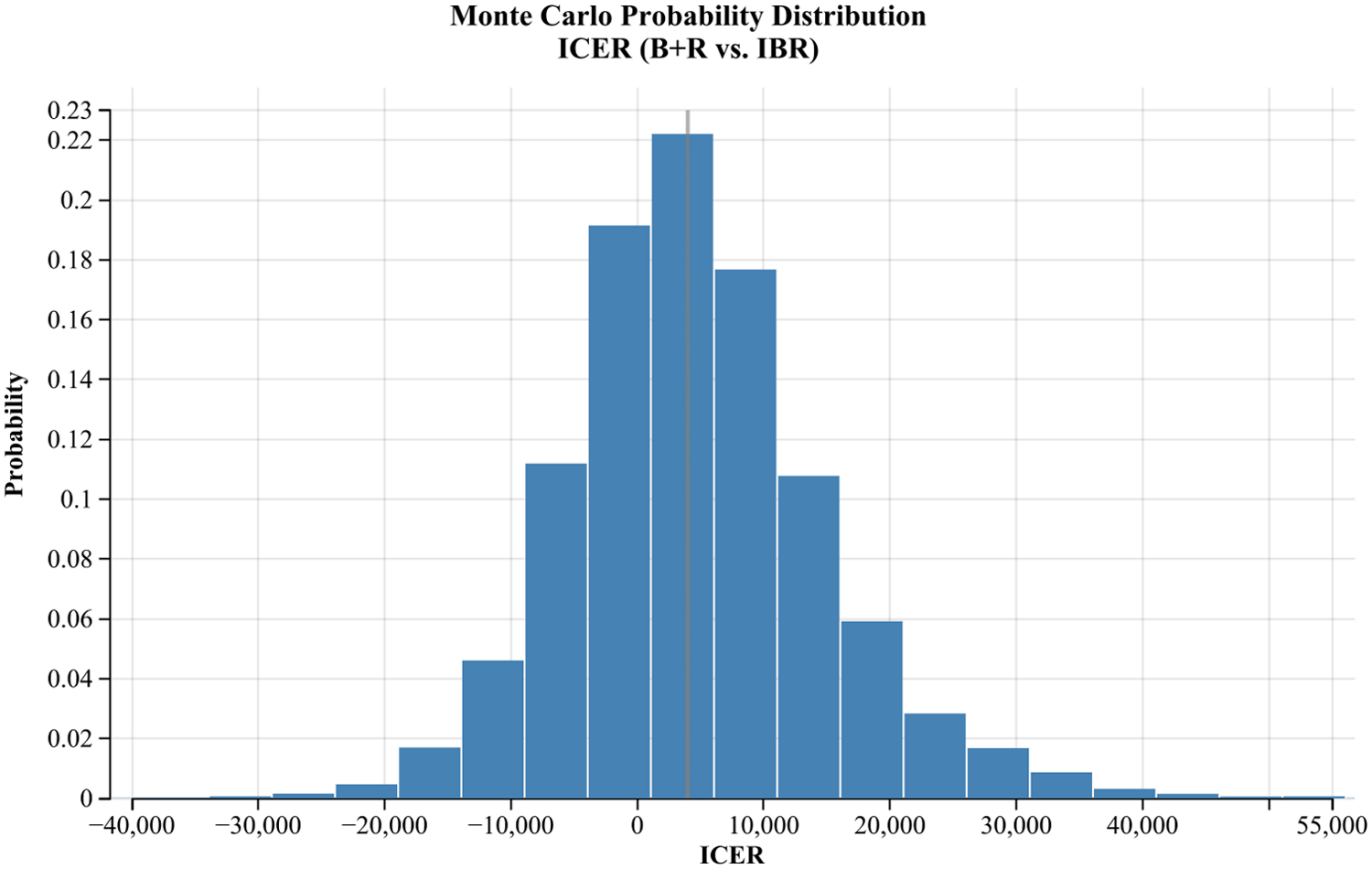
Histogram of incremental cost-effectiveness ratios (ICERs) comparing Bankart repair with remplissage (BR+R) with isolated Bankart repair (IBR). The majority of ICERs fell between $0 and $20,000 per quality-adjusted life-year (QALY), with a mean of $3977. Less than 1% of ICERs exceeded the willingness-to-pay (WTP) threshold of $50,000/QALY.

The distribution of ICERs across the 10,000 PSA iterations is shown in Figure 4. The majority of ICERs fell between $0/QALY and $20,000/QALY, with the concentration of values below the WTP threshold of $50,000. A proportion of ICERs fell below zero, representing simulations in which BR+R was both more effective and less costly than IBR. BR+R was dominant, yielding both higher QALYs and lower total costs than IBR, in 32.99% of 10,000 PSA iterations.

**Figure 5.**
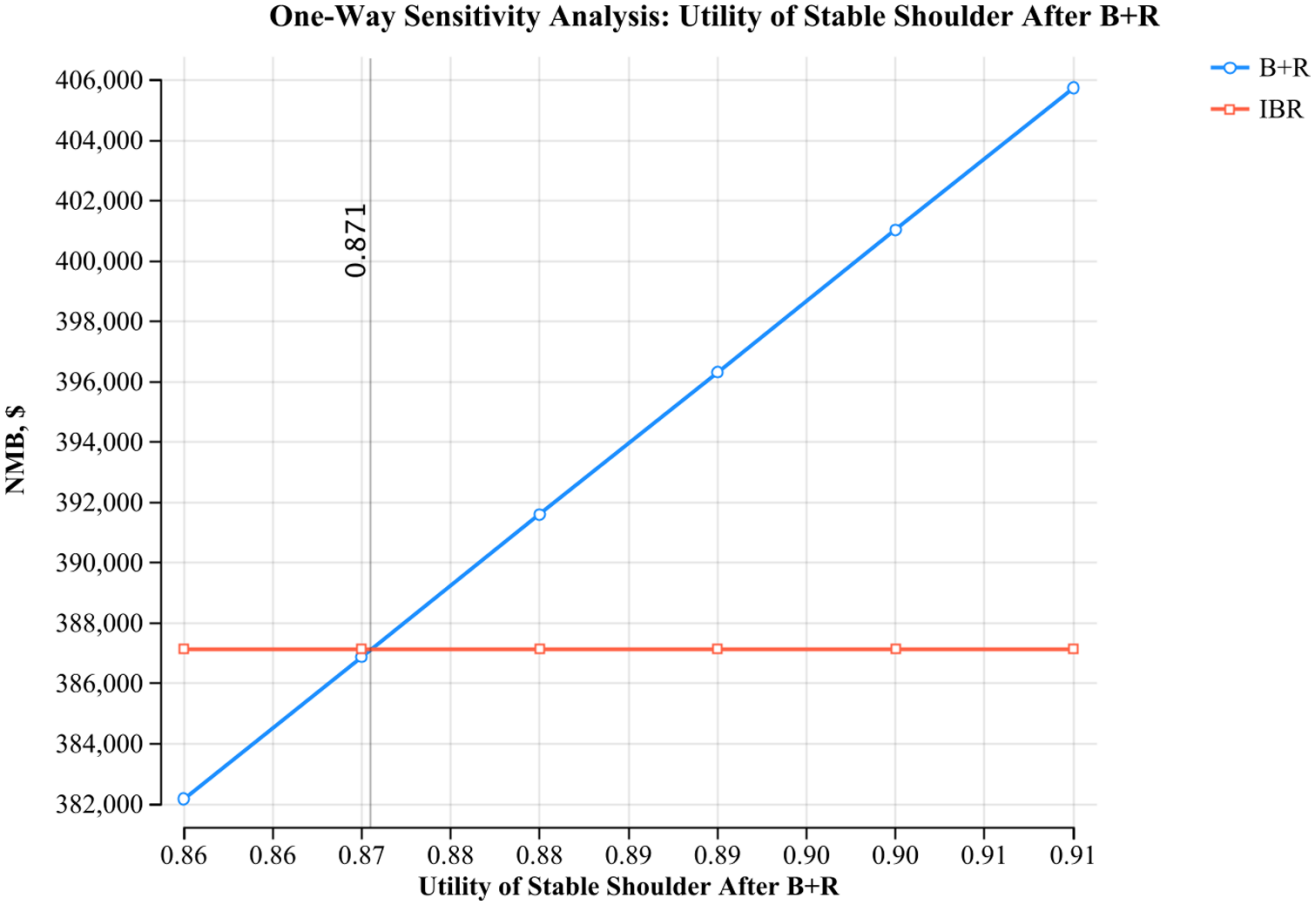
One-way sensitivity analysis of the utility assigned to the stable shoulder state after Bankart repair with remplissage (BR+R), expressed as a net monetary benefit (NMB) at a willingness-to-pay (WTP) threshold of $50,000 per quality-adjusted life-year (QALY). The range-of-motion (ROM) loss decrement varied from 0.00 to 0.05 (effective utility range, 0.91-0.86). BR+R remained the preferred strategy until the utility fell below 0.871, corresponding to a ROM-related utility decrement of 0.039.

One-way sensitivity analysis showed that model outcomes were most sensitive to recurrence probabilities. BR+R remained the preferred strategy unless the recurrence probability for IBR during years 1-3 fell below 0.30% (base case: 4.00%) or the recurrence probability for BR+R during years 1-3 exceeded 4.45% (base case: 0.62%). BR+R also remained cost-effective unless the utility assigned to recurrent instability exceeded 0.873 (base case: 0.35) or the BR+R procedure cost exceeded $36,646 (base case: $14,550). One-way sensitivity analysis on the utility of the stable shoulder state after BR+R, in which the ROM loss decrement varied from 0.00 to 0.05 (effective utility range, 0.91-0.86), demonstrated that BR+R remained cost-effective until this utility fell below 0.871 at a WTP threshold of $50,000/QALY, corresponding to a ROM-related decrement of 0.039 from the base-case stable shoulder utility of 0.91 (Figure 5). At a WTP threshold of $100,000/QALY, this decreased to 0.869, corresponding to a decrement of 0.041.

## Discussion

The results of this Markov decision analytic model indicate that Bankart repair with remplissage was more cost-effective than isolated Bankart repair for anterior shoulder instability with on-track Hill-Sachs lesions and subcritical GBL. The mean ICER of $3977 per QALY gained was well below standard WTP thresholds of $50,000/QALY and $100,000/QALY. Despite a modest incremental cost of $1646 for BR+R over the 10-year time horizon, this strategy was preferred in 99.89% of PSA iterations at a WTP threshold of $50,000 and in 99.99% of simulations at $100,000, with findings consistent with one-way sensitivity analysis. In 32.99% of simulations, BR+R was dominant, yielding both higher QALYs and lower total costs, reflecting that reductions in revision surgery frequently offset the higher upfront procedure cost of remplissage. The incremental QALY gain of 0.41, equivalent in value to approximately 5 additional months of perfect health, was the main contributor to cost-effectiveness rather than direct cost savings. These findings support the hypothesis that BR+R is cost-effective for on-track Hill-Sachs lesions, driven primarily by reduced recurrent instability events with the addition of remplissage.

One-way sensitivity analysis demonstrated that all threshold values required to change the preferred strategy fell well outside clinically plausible ranges. The model was most sensitive to recurrence probabilities, but the IBR recurrence rate during years 1-3 would need to fall below 0.30% and the BR+R recurrence rate would need to exceed 4.45%, both of which are unsupported by any published data on this population. The BR+R procedure cost of $36,646 was more than 2.5 times the base-case procedure cost of $14,550, underscoring that cost-effectiveness is maintained even under extreme cost assumptions. The recurrent instability utility of 0.873 is also notable because it substantially exceeded the base-case value of 0.35, suggesting that even if the utility penalty for recurrent instability is far less severe than estimated from WOSI-derived data, BR+R remains preferred.

This study builds upon recent economic analyses of remplissage by Schreiner et al,^43^ who used break-even analysis to show that remplissage is economically justified for off-track lesions and variably financially viable for on-track lesions depending on institutional cost parameters. Their analysis demonstrated that even modest reductions in recurrence rates with remplissage can translate to meaningful cost savings by avoiding revision surgery, particularly given the relatively low incremental cost of adding remplissage to the index procedure. However, break-even analyses do not capture quality-of-life differences between treatment strategies or model outcomes over extended time horizons. Our Markov model addressed these limitations by incorporating health utilities over a 10-year period, showing that QALY gains from reduced recurrent instability substantially contributed to the value of remplissage beyond direct cost considerations alone. Additionally, by adopting a societal perspective, the present analysis captured indirect costs related to productivity loss, as each avoided revision surgical procedure also avoids weeks of missed work time.

While our findings demonstrate that remplissage is cost-effective from a societal perspective, practical barriers to adoption warrant consideration. Remplissage requires additional operative time and suture anchors, increasing the direct procedure cost relative to IBR.^43^ Despite this, there is no dedicated Current Procedural Terminology (CPT) code for remplissage. The most commonly used code, CPT 29806, is already used for Bankart repair and broadly describes arthroscopic capsulolabral repair, which may technically encompass remplissage as a form of capsulolabral reconstruction. Alternatively, surgeons may bill remplissage under CPT 29999, an unlisted arthroscopic procedure code, although reimbursement under this code is variable and often requires additional justification. However, because neither code was designed to capture the added work of a separate Hill-Sachs defect–filling procedure, it remains unclear what the appropriate relative value units (RVUs) should be for the addition of remplissage. This may result in inconsistent and inadequate reimbursement, which is particularly relevant in the context of steadily declining orthopaedic surgeon reimbursement. Our findings, showing that the addition of remplissage yielded a favorable ICER well below standard WTP thresholds and reduced downstream revision surgery costs, provide economic evidence that could support advocacy for appropriate RVU valuation or a distinct procedural code for remplissage. Establishing a clear reimbursement framework may help align payer incentives with the long-term value demonstrated by this procedure.

The clinical inputs for this model were informed by a recent systematic review by Villarreal-Espinosa et al,^50^ who reported lower ranges of recurrent instability rates with BR+R compared with IBR in 537 patients with on-track Hill-Sachs lesions and subcritical GBL. Across the included studies, recurrent dislocation rates ranged from 0% to 7.7% with BR+R versus 3.5% to 30% with IBR, with significantly lower odds of recurrence with BR+R (OR, 0.22 [95% CI, 0.09-0.53]). This protective effect was maintained in high-risk subgroups, including contact athletes (OR, 0.23 [95% CI, 0.07-0.83]) and patients with near-track lesions (OR, 0.28 [95% CI, 0.10-0.78]), who have an elevated baseline recurrence risk with IBR alone. These findings challenge the traditional approach to on-track lesions, suggesting that more frequent utilization of remplissage in this population may be appropriate. Because our model used pooled recurrence rates across all patients with on-track lesions, it likely represents a conservative estimate of cost-effectiveness for these high-risk subgroups in which the benefit of remplissage may be even greater.

This aligns with a growing body of evidence that the binary approach to on-track and off-track Hill-Sachs lesions oversimplifies a continuum of risk. Yamamoto et al^55^ introduced the concept that on-track lesions exist on a spectrum, demonstrating that patients with “peripheral-track” lesions have significantly worse outcomes, despite being classified as on-track. Subsequent studies have introduced the “distance to dislocation” (DTD) metric, defined as the difference between the glenoid track width and Hill-Sachs interval, and found that on-track lesions with a DTD <8 to 10 mm predicted significantly higher failure rates after IBR.^3,27^ Notably, collision athletes maintain an elevated recurrence risk (>12%) even at DTD values up to 24 mm, suggesting that these patients may benefit from remplissage augmentation, regardless of track status.^3^ Recent clinical studies have further supported the benefit of remplissage in higher risk patients with on-track lesions. IBR has been shown to carry an approximately 8-fold higher risk of recurrent instability compared with remplissage augmentation in patients with multiple risk factors including younger age, contact sport participation, hyperlaxity, and near-track lesions.^9^ Similarly, remplissage significantly reduced recurrence in patients with critical humeral bone loss, defined as inferior Hill-Sachs extension >90° on sagittal magnetic resonance imaging.^47^ Combined with the present cost-effectiveness findings, this growing body of clinical evidence supports more frequent consideration of remplissage in patients with on-track Hill-Sachs lesions, particularly in those at a higher risk for recurrence.

The cost-effectiveness of remplissage is likely influenced by patient-specific risk factors. Because our model used pooled recurrence rates across all patients with on-track lesions, the results represent an average risk estimate. Patients with an elevated baseline recurrence risk, including younger patients, contact athletes, those with hyperlaxity, and those with near-track lesions, would be expected to derive a greater benefit from remplissage, amplifying the incremental reduction in recurrence and downstream revision surgery that drove cost-effectiveness in this model. Conversely, the economic benefit may be smaller in lower risk patients or in those for whom ROM preservation is critical, such as overhead or throwing athletes, in which lower return-to-sport rates after remplissage have been reported.^21^ Surgeon-level factors, including familiarity with the remplissage technique and institutional cost structures, may also influence the practical value of adding remplissage in individual settings. Further prospective studies in high-risk on-track subgroups will help refine patient selection and optimize the role of remplissage.

Several potential downsides to remplissage merit consideration. Although biomechanical studies have demonstrated decreased external rotation after remplissage, clinical studies generally show smaller differences that are often not clinically meaningful and may diminish with longer follow-up.^1,15,31,54^ Caution is warranted in throwing athletes, who demonstrate lower return-to-sport rates of 46% to 79% compared with contact athletes (80%-100%), thought to reflect the sport-specific demands of external rotation.^21^ In the present model, a one-way sensitivity analysis applied a conservative ROM loss decrement utility, varied from 0.00 to 0.05, to the BR+R stable shoulder state to account for the theoretical concern of reduced external rotation after remplissage. The analysis demonstrated that BR+R remained cost-effective unless this decrement exceeded 0.039, a threshold well beyond what current clinical evidence supports.^1,31^ Regarding osteoarthritis, biomechanical studies suggest that remplissage may alter joint mechanics in ways that could affect articular cartilage over time,^2,18,54^ but clinical evidence of accelerated osteoarthritis progression has not been demonstrated. Any such effect would likely manifest beyond the 10-year time horizon of this model and is therefore not captured in our analysis.

This study has several limitations. Randomized controlled trial data comparing BR+R and IBR for on-track Hill-Sachs lesions are not available; thus, transition probabilities were derived from random-effects meta-analysis of level 3 comparative studies included in the systematic review by Villarreal-Espinosa et al.^50^ The authors of that study did not pool recurrence rates because of heterogeneity and the risk of bias among the included studies, but pooled estimates were necessary to parameterize a Markov model. Heterogeneity was addressed through the use of random-effects meta-analysis and empirically derived variance estimates in PSA. Among the included studies, GBL ranged from 2.7% to 20%, potentially introducing additional heterogeneity.^50^ The extrapolation of cumulative recurrence rates to period-specific annual probabilities relied on the temporal distribution reported by Do et al,^16^ which may not precisely reflect the temporal pattern of the recurrence risk. The model limited patients to a maximum of 2 surgical procedures (index procedure and revision Latarjet procedure), similar to prior cost-effectiveness models that capped the number of revision procedures.^28,33^ This assumption was applied equally to both treatment arms, and because the revision Latarjet procedure was modeled identically for both arms, any additional revisions would occur at equal rates. Furthermore, because preventing primary recurrence reduces downstream revision events, allowing additional revisions would be expected to favor BR+R. Utilities were modeled by stability state and may not fully capture sport-specific performance deficits related to postoperative ROM, although sensitivity analysis on ROM-related utility decrements demonstrated that BR+R remained cost-effective across a wide range of assumptions.

## Conclusion

For patients with on-track Hill-Sachs lesions and subcritical GBL, Bankart repair with remplissage was cost-effective compared with isolated Bankart repair, driven by reductions in recurrence and revision surgery. These findings support the consideration of remplissage in patients with on-track lesions, particularly those at a higher risk for recurrence.
